# Emotion regulation and mental well-being before and six months after bariatric surgery

**DOI:** 10.1007/s40519-017-0379-8

**Published:** 2017-04-07

**Authors:** Christiane Efferdinger, Dorothea König, Alexander Klaus, Reinhold Jagsch

**Affiliations:** 1 Department of Applied Psychology: Health, Development, Enhancement, Intervention, Faculty of Psychology, University of Vienna, Liebiggasse 5, 1010 Vienna, Austria; 2 Krankenhaus der Barmherzigen Schwestern, Stumpergasse 13, 1060 Vienna, Austria

**Keywords:** Bariatric surgery, Emotion regulation, Mental well-being, Depressive symptoms, Health-related quality of life

## Abstract

**Purpose:**

According to the current state of research, mental health improves due to bariatric surgery. However, improvements in weight and psychosocial aspects often show a gradual decline with time. As emotion regulation (ER) appears to be a key variable in the successful outcome of weight loss treatments, the present study aimed at investigating ER-strategies applied by bariatric surgery candidates pre- and post-surgery and examining interactions between ER, depressive symptoms, health-related quality of life (HrQoL), and post-surgical weight loss.

**Methods:**

Prior to and 6 months after bariatric surgery, 45 patients (76% women) completed self-report questionnaires assessing depressive symptoms (Beck Depression Inventory-II), HrQoL (Short Form-36 Health Survey), and ER-strategies (Emotion Regulation Inventory for Negative Emotions).

**Results:**

Six months post-surgery, the patients reported significant improvements in depressive symptomatology, HrQoL, and satisfaction with ER compared to pre-surgery. Groups differing in their course of ER-satisfaction also differed in psychosocial dimensions pre- to post-surgery, increased satisfaction being related to less impairment and enhanced communication of negative emotions as a form of an adaptive regulation. Patients with higher weight loss applied the strategy of controlled expression more frequently post-surgery than pre-surgery and compared to patients with lower weight loss.

**Conclusions:**

Postoperative weight loss leads to improvements in ER-satisfaction and mental well-being. As satisfaction with ER seems to be associated with less impaired mental well-being among bariatric surgery candidates, presumably even more positive psychosocial outcomes could be obtained post-surgery by implementing trainings explicitly encouraging the use of adaptive ER-strategies.

## Introduction

Morbid obesity is increasing faster than any other disease worldwide. It is associated with an elevated risk of morbidity and mortality as well as impaired mental well-being [1]. Candidates for bariatric surgery are particularly afflicted, with research indicating that up to two-thirds of the patients suffer from one or more Axis I disorders [2, 3]. Affective disorders are the most commonly reported disorders, with a lifetime prevalence of 55% [4]—a percentage that rivals the prevalence of most of the major medical comorbidities among bariatric patients. Patients requiring bariatric surgery also consistently report a poor health-related quality of life (HrQoL), the physical components, measured with the Short Form-36 Health Survey (SF-36), being more impaired than the mental components [5, 6].

To date, bariatric surgery is the only proven treatment that achieves sustained weight loss in the majority of morbidly obese patients [7]. Meta-analyses provide evidence for the positive outcomes of bariatric surgery on numerous medical conditions [8, 9] and aspects of mental health [10]. Mack et al. [11] and Aasprang et al. [12] found significant improvements of depressive symptoms and HrQoL as they examined patients up to 5 years after surgery. Peak improvements are usually reported 1 year after surgery, corresponding to the initial course of weight loss. However, as patients begin to regain weight over time, improvements in mental well-being gradually decline as well [13–15]. Nevertheless, weight, depressive symptoms, and the physical dimension of HrQoL still remain substantially improved in the long term, whereas the mental dimension of HrQoL appears to decline with time [13], sometimes even reaching baseline again [16]. Some researchers, however, propose that psychosocial factors do not worsen because of weight regain but, vice versa, impairments in mental well-being contribute to an unsuccessful weight loss or weight regain at some point post-surgery [17, 18]. Either way, emotion regulation (ER) seems to be a crucial factor in the successful outcome of weight loss treatments [19].

Deficits in ER play an important role in psychopathology [20, 21] such as affective disorders [22] and eating disorders [23–25]. Binge eating disorder, highly prevalent among obese individuals with a rate twice to five times as high as in the population with BMI <30 kg/m² [26], was found to be related to maladaptive ER-strategies such as suppression of emotions [24, 25]. Non-acceptance of emotional responses emerged as a strong predictor of general eating pathology in obese individuals with binge eating disorder [27] and was recently found to play a role in non-normative eating behaviors such as emotional eating in a preoperative sample [28]. Baldofski et al. [28] state that obese individuals are likely to experience strong negative emotions due to the internalization of weight-based stereotypes while at the same time they seem to lack adaptive ER-strategies, hence engaging in emotional eating and binge eating as a way of dealing with emotions.

Given the high prevalence of affective disorders [4, 10, 29] as well as non-normative eating behaviors and binge eating (disorder) among obese individuals [26, 27] and in particular among bariatric surgery candidates [10, 30–33], each of which is known to be associated with difficulties in ER, further research in this respect is required. With the help of new findings, psychological interventions focusing on ER could be implemented and specifically adapted to meet the needs of obese individuals [34, 35], supporting patients both before and after bariatric surgery.

The main focus of the present study therefore lays on determining ER-strategies applied by bariatric surgery candidates pre- and post-surgery and providing information on interactions between ER, depressive symptoms, HrQoL, and post-surgical weight loss.

## Methods

### Participants

Candidates for bariatric surgery were patients with a BMI ≥40 or ≥35 kg/m² with severe obesity-associated medical conditions. Each surgical candidate underwent a multidisciplinary evaluation. Patients approved for surgery had no medical and psychological contraindications for bariatric surgery. Subjects were excluded from the study if they had previously had bariatric surgery.

### Measures

The Beck Depression Inventory-II (BDI-II) [36] is a 21-item survey designed to assess the severity of depressive symptomatology during the last two weeks. Sum scores range between 0 and 63. The severity ranges are as follows: 0–13, minimal depression; 14–19, mild depression; 20–28, moderate depression; and 29–63, severe depression [36]. Internal consistency (Cronbach’s alpha) in the present sample was excellent (α = 0.91).

To assess HrQoL, the German version of the Short Form-36 Health Survey (SF-36) was used [37]. The SF-36 is a generic measure consisting of the following eight subscales (scores: 0–100): physical functioning, role-physical, bodily pain, general health, vitality, social functioning, role-emotional, and mental health. The physical component summary score (PCS) was computed as the mean of the first four subscales (α = 0.85), and the mental component summary score (MCS) as the mean of the four latter subscales (α = 0.84).

ER-strategies were assessed using the Emotion Regulation Inventory for Negative Emotions (ERI-NE) [38]. This 22-item questionnaire is composed of the following five scales (scores: 0–100): distraction (distracting oneself), reappraisal (seeing the situation/emotion in a more positive light), empathic suppression (suppressing emotions so as not to impose on others), uncontrolled expression (expressing emotions unrestrainedly), and controlled expression (communicating emotions). In this study, internal consistency for the scales was good to excellent (0.81 ≤ α ≤0.97). An additional item refers to the satisfaction with the own regulation of negative emotions (1 = very unsatisfied to 7 = very satisfied).

Higher scores in the measures indicate a more pronounced depressive symptomatology, better HrQoL, more frequent use of ER-strategies, and higher ER-satisfaction, respectively.

### Procedure

A pre-test/post-test design was used. All subjects were given questionnaires assessing depressive symptoms, HrQoL, and ER-strategies the day before surgery. Six months post-surgery, the questionnaires were mailed to the patients again, including a covering letter and a self-addressed stamped envelope.

### Statistical analysis

Data were analyzed using SPSS 22. Total percent weight loss was calculated as (weight loss (kg)/weight pre-surgery (kg) × 100) and percent excess weight loss (EWL) as (weight loss (kg)/excess weight (kg) × 100). Statistical significance was defined as *p* < 0.05. To compare changes in the psychosocial constructs pre- to post-surgery, dependent-samples *t* tests were performed. To evaluate the effect of differences between two groups, Cohen’s *d* was calculated, with values ≥0.20 indicating a small, ≥0.50 a medium, and ≥0.80 a large effect. General linear models (GLM) for repeated measures were applied in order to establish whether groups differing in their course of ER-satisfaction also differed in the psychosocial constructs over time. Post hoc tests (Scheffé) were calculated for subscales revealing a significant group main effect. GLM for repeated measures were also conducted to analyze possible differences in the psychosocial constructs over time depending on the amount of weight loss (two groups according to EWL ≥50% or <50%). To evaluate the effect of differences identified by means of GLM, partial eta^2^ (η_p_^2^) was calculated, with values ≥0.01 indicating a small, ≥0.06 a medium, and ≥0.14 a large effect. For controlling the alpha error caused by multiple testing, the Bonferroni–Holm method was used.

## Results

Sixty-six patients completed the questionnaires pre-surgery and 45 of them (68%) also completed the questionnaires post-surgery. Statistical comparisons of preoperative data (demographics, BMI, level of depression, HrQoL, and ER-strategies used) between the subset of patients who did not respond at post-surgery and those who responded demonstrated no significant differences. The responder sample (*n* = 45) had a mean age of 44.07 years (±13.28, range 20–68 years) and comprised 34 (76%) females; 96% identified themselves as Austrian. The mean BMI pre-surgery was 45.59 kg/m² (±7.45, BMI ranging from 35.35 to 65.86 kg/m²). Roux-en-Y gastric bypass was performed in 33 cases (73%), and sleeve resection was carried out in 12 cases (27%). Six months post-surgery, patients’ mean BMI was 33.65 kg/m² (±6.98), ranging from 23.83 to 56.61 kg/m², indicating a significant BMI decrease pre- to post-surgery [*t*(43) = 24.604, *p* < 0.001, *d* = 1.63]. On average, patients lost 26% (±7, range 13–40%) of their initial weight; mean EWL was 62% (±19, range 21–109%).

Regarding depressive symptomatology and HrQoL, we found significant improvements pre- to post-surgery with large effects (see Table 1). While patients on average reported a mild depression pre-surgery (BDI-II: 17.00 ± 12.05), the mean postoperative BDI-II score (6.53 ± 7.96) corresponded to a minimal depressive symptomatology. In terms of HrQoL, the MCS was found to be significantly lower than the PCS [*t*(41) = 2.739, *p* = 0.009, *d* = 0.30] within the postoperative sample, while no significant differences were found within the preoperative sample [*t*(41) = −1.039, *p* = 0.305].

**Table 1:** Descriptive statistics (*M* ± SD) for psychosocial constructs and test statistics for group comparisons pre- versus post-surgery (dependent-samples *t* tests)

Measures	Subscales	Pre-surgery (*n* = 45)	Post-surgery (*n* = 45)	*t*	df	*p*	*p*_adj_	*d*
BDI-II		17.00 ± 12.05	6.53 ± 7.96	6.362	44	<0.001	0.0063	0.99
SF-36	Physical component summary score	54.75 ± 25.77	84.58 ± 14.27	−7.309	38	<0.001	0.0056	1.39
	Mental component summary score	57.74 ± 25.24	80.17 ± 16.85	−5.791	41	<0.001	0.0071	1.03
ERI-NE	Distraction	57.92 ± 19.83	61.25 ± 22.64	−0.939	44	0.353	0.0167	0.16
	Reappraisal	56.84 ± 20.97	59.31 ± 23.48	−0.747	44	0.459	0.0250	0.11
	Empathic suppression	57.36 ± 19.41	51.25 ± 27.00	1.393	44	0.170	0.0125	0.26
	Uncontrolled expression	26.67 ± 16.55	28.00 ± 20.46	−0.502	44	0.618	0.0500	0.07
	Controlled expression	50.54 ± 27.16	59.89 ± 33.12	−1.796	44	0.079	0.0100	0.31
	Satisfaction with emotion regulation	4.60 ± 1.71	5.64 ± 1.15	−4.175	44	<0.001	0.0083	0.70

*BDI-II* Beck Depression Inventory-II, *SF-36* Short Form-36 Health Survey (varying *df* due to missing data), *ERI-NE* Emotion Regulation Inventory for Negative Emotions, *p*_adj_ Bonferroni–Holm adjusted *p* scores, *d* effect size Cohen’s *d*

Regarding the use of ER-strategies, no statistically significant improvements were found pre- to post-surgery. However, 6 months post-surgery the patients were significantly more satisfied with their ER than pre-surgery, with *d* = 0.70 indicating a medium effect.

Examining the significant improvement in ER-satisfaction more closely, three groups of patients could be identified: the *positive change* group (*n* = 14) was unsatisfied pre-surgery, but satisfied post-surgery; about half of the sample (*n* = 23) was satisfied both pre- and post-surgery (*stable positive*); the smallest group (*n* = 8) showed *no positive change* with regard to their ER-satisfaction over time, hence remaining unsatisfied.

**Fig. 1:**
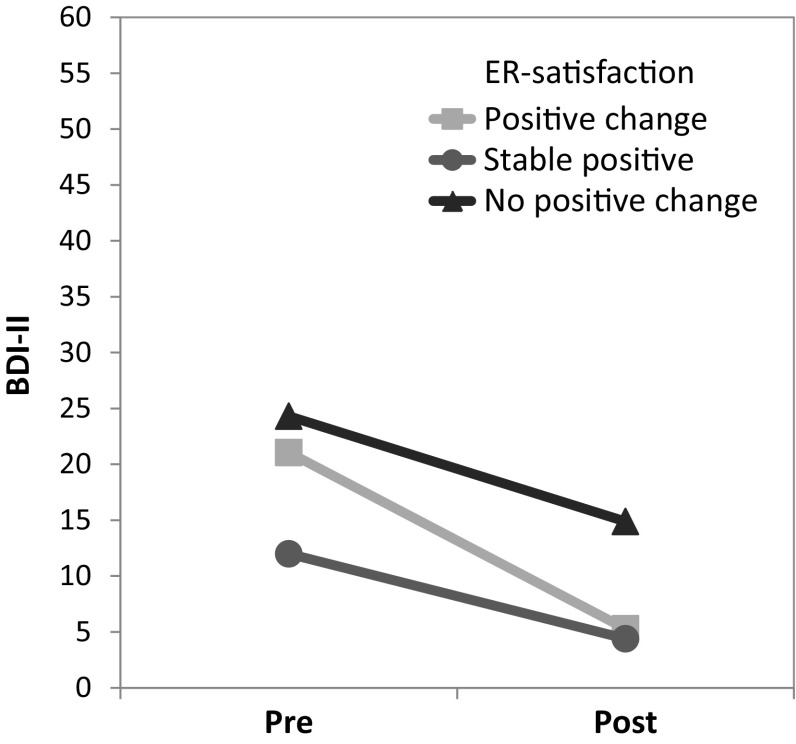
Beck Depression Inventory-II (BDI-II) scores pre- and post-surgery for three groups according to satisfaction with emotion regulation (ER). Time main effect: *p* < 0.001; group main effect: *p* = 0.002, post hoc tests (Scheffé): no positive change versus stable positive (*p* = 0.003)

To compare these three groups in regard to psychosocial constructs pre- to post-surgery, 2 × 3 GLM were applied (see Table 2). In terms of depression, a significant time main effect (*p* < 0.001) as well as a significant group main effect (*p* = 0.002) could be observed. Post hoc tests revealed differences between the groups *stable positive* and *no positive change* (*p* = 0.003). Figure 1 indicates a considerable decrease in the depression score over time, especially within the *positive change* group (*p* = 0.002); however, the interaction of time and group did not reach statistical significance (*p* = 0.086). For both, the PCS and the MCS, significant time main effects were found (*p*s < 0.001). Additionally, the *stable positive* group showed a significantly higher MCS than the *no positive change* group (*p* = 0.001).

**Table 2:** Descriptive statistics (*M* ± SD) for psychosocial constructs pre- and post-surgery classified in three groups according to satisfaction with emotion regulation (ER), and test statistics for 2 × 3 general linear models (GLM)

Measures	Subscales	Positive change (*n* = 14)	Stable positive (*n* = 23)	No positive change (*n* = 8)	*Time* main effect			*Group* main effect			Interaction			*p*_adj_
					*F*	*p*	η_p_^2^	*F*	*p*	η_p_^2^	*F*	*p*	η_p_^2^	
BDI-II		21.05 ± 13.12	12.00 ± 9.22	24.30 ± 12.23	39.466	<0.001	0.484	6.984	0.002^a^	0.250	2.598	0.086	0.110	0.0071
		5.29 ± 3.22	4.39 ± 6.72	14.88 ± 11.79										
SF-36	PCS	48.44 ± 26.38	60.96 ± 24.49	45.63 ± 27.55	43.796	<0.001	0.549	2.356	0.109	0.116	1.827	0.176	0.092	0.0083
		89.71 ± 5.42	85.99 ± 12.45	69.42 ± 22.74										
	MCS	50.00 ± 23.24	68.08 ± 21.19	39.61 ± 28.20	29.947	<0.001	0.434	9.058	0.001^b^	0.317	1.319	0.279	0.063	0.0100
		81.54 ± 8.15	85.51 ± 13.19	60.86 ± 25.59										
ERI-NE	Distraction	56.25 ± 20.36	61.14 ± 20.20	51.56 ± 18.22	0.651	0.424	0.015	1.131	0.332	0.051	0.573	0.568	0.027	0.0500
		65.18 ± 15.45	62.50 ± 26.58	50.78 ± 19.89										
	Reappraisal	46.43 ± 16.57	66.65 ± 19.94	46.88 ± 18.90	0.427	0.517	0.010	5.996	0.005^c^	0.222	0.763	0.473	0.035	0.0250
		54.91 ± 24.17	66.85 ± 22.09	45.31 ± 19.97										
	Empathic suppression	54.46 ± 21.57	57.61 ± 18.07	61.72 ± 20.98	0.434	0.513	0.010	1.789	0.180	0.079	1.147	0.327	0.052	0.0167
		49.11 ± 21.77	46.47 ± 27.81	68.75 ± 28.93										
	Uncontrolled expression	30.00 ± 16.87	25.65 ± 18.67	23.75 ± 7.91	1.118	0.296	0.026	0.991	0.380	0.045	1.248	0.297	0.056	0.0125
		34.29 ± 19.99	23.04 ± 17.43	31.25 ± 27.61										
	Controlled expression	41.79 ± 28.53	63.45 ± 19.42	28.73 ± 26.39	3.516	0.068	0.077	7.061	0.002^d^	0.252	3.006	0.060	0.125	0.0063
		69.29 ± 32.28	64.13 ± 31.14	31.25 ± 27.22										

For each measure/subscale, *M* ± SD pre-surgery presented in the first row, *M* ± SD post-surgery presented in the second row; *BDI-II* Beck Depression Inventory-II; *SF-36* Short Form-36 Health Survey; *PCS* Physical component summary score; *MCS* Mental component summary score; *ERI-NE* Emotion Regulation Inventory for Negative Emotions; η_p_^2^ effect size partial eta^2^; *p*_adj_ Bonferroni–Holm adjusted *p* scores

^a^Post hoc tests (Scheffé) BDI-II: stable positive versus no positive change: *p* = 0.003

^b^Post hoc tests (Scheffé) MCS: stable positive versus no positive change: *p* = 0.001

^c^Post hoc tests (Scheffé) Reappraisal: positive change versus stable positive: *p* = 0.033, stable positive versus no positive change: *p* = 0.022

^d^Post hoc tests (Scheffé) Controlled expression: positive change versus no positive change: *p* = 0.041, stable positive versus no positive change: *p* = 0.002

**Fig. 2:**
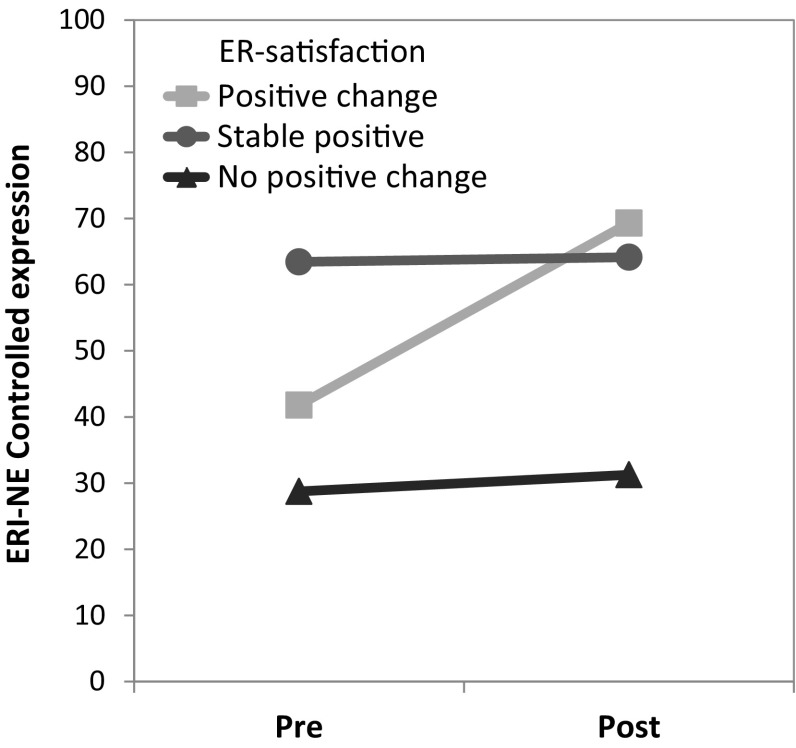
Controlled expression scores pre- and post-surgery for three groups according to satisfaction with emotion regulation (ER). *ERI-NE* Emotion Regulation Inventory for Negative Emotions. Group main effect: *p* = 0.002, post hoc tests (Scheffé): no positive change versus stable positive (*p* = 0.002) and positive change (*p* = 0.041)

Varying results were obtained regarding ER-strategies. Significant effects were found for neither *distraction* and *empathic suppression* nor *uncontrolled expression*. In case of *reappraisal*, the group main effect was significant (*p* = 0.005), displaying high scores for the *stable positive* group which significantly differed from the other two groups according to post hoc tests. Regarding *controlled expression*, the significant group main effect (*p* = 0.002) and applied post hoc tests indicated that individuals *without positive change* of ER-satisfaction communicated negative emotions less frequently than the other two groups. Pre- to post-surgery, the use of *controlled expression* significantly increased in the *positive change* group only (*p* = 0.013). The interaction of time and group, however, was non-significant (*p* = 0.060) (Fig. 2).

In order to compare psychosocial changes over time depending on the amount of weight loss, the sample (one missing value) was divided into patients with an EWL ≥50% (*n* = 29, 66%) and patients with an EWL <50% (*n* = 15, 34%). To establish whether patients with a higher weight loss also showed greater psychosocial improvements over time, the two EWL groups were compared using GLM for repeated measures. Patients’ scores of depressive symptomatology and of all HrQoL subscales significantly improved over time (all *p*s < 0.001) with huge effects (η_p_²) ranging from 0.26 to 0.75. Results, however, showed no significant differences between the two EWL groups in terms of depressive symptomatology and HrQoL, indicating that patients with less weight loss had equally benefited from bariatric surgery as patients with a higher EWL. A significant interaction effect was found for the scale *controlled expression* (*F*(1, 42) = 4.268, *p* = 0.045, η_p_² = 0.09 indicating a medium effect). Patients with an EWL ≥50% made more use of the strategy post-surgery (68.45 ± 30.48) compared to pre-surgery (51.38 ± 26.66), while the opposite trend was found for patients with an EWL <50% (42.33 ± 33.05 post-surgery, 47.63 ± 29.33 pre-surgery). Post-surgery, patients with EWL ≥50% applied the strategy more frequently than patients with EWL <50% [*t*(42) = 2.619, *p* = 0.012, *d* = 0.83 indicating a large effect].

## Discussion

Patients reported severe impairments in depressive symptomatology and HrQoL pre-surgery. Six months after bariatric surgery, their mental well-being had considerably improved. Individuals reported significantly fewer depressive symptoms and improvements in HrQoL; however, the MCS of the SF-36 was found to be lower than the PCS post-surgery. While studies indicate a deterioration of the MCS with time after an initial improvement [13, 16], the present study suggests that patients already find it harder to recover from the psychosocial than physical consequences of obesity in the first place. Yet, a considerable improvement was shown in both the physical and the psychosocial dimensions 6 months after surgery.

Patients reported a considerable improvement of satisfaction with ER over time. However, with regard to the course of ER-satisfaction over time, three different groups (*positive change, stable positive*, and *no positive change*) could be identified. Group comparisons in regard to psychosocial constructs over time suggest that higher ER-satisfaction (stable or positively changing) is associated with more favorable psychosocial outcomes 6 months post-surgery (e.g., less depressive symptomatology and increased use of controlled expression to regulate negative emotions in an adaptive way). Enhancing ER-satisfaction might therefore be a promising approach in future weight loss treatments—training patients in ER, thereby encouraging their confidence in being able to successfully control emotions and apply adaptive ER-strategies effectively [39], might contribute to improving ER-satisfaction.

Findings suggest that individuals with a higher EWL develop a more adaptive way of dealing with negative feelings post-surgery. Patients with EWL ≥50% increasingly regulated their emotions by talking about them—an effect that could not be found for patients with EWL <50%. Presumably, individuals with a higher weight loss feel less ashamed and shy in social situations, finding it easier to share and communicate their feelings.

While these results indicate that massive weight loss due to bariatric surgery itself already leads to improvements in ER-satisfaction and hence mental well-being, it seems very likely that even better results could be obtained by implementing additional ER-trainings. Recent pilot studies provide evidence that psychological interventions with an ER-focus in the course of bariatric services achieve beneficial outcomes such as a decrease in depressive symptoms and eating disorders and successful weight loss [34, 35]. A positive impact of group-based cognitive behavioral therapy (CBT) on weight loss [40] and binge eating [41] in bariatric surgery candidates has already been demonstrated. In an 8-week CBT-based group, including elements of ER, significant improvements of various psychological outcomes (e.g., frequency of anxiety and depressive symptoms, perceived difficulties in life, weight-related adjustment) have recently been recorded post-surgery [17], possibly setting the direction for further research on the topic of ER-interventions among bariatric surgery candidates.

Need for systematic interventions is high as a study by de Zwaan et al. [31] demonstrated that 25% of the participants reported “loss of control” eating on average 2 years after bariatric surgery, associated with impaired mental well-being and poorer weight loss. A preoperative binge eating disorder turned out to be a significant risk factor for the development of non-normative eating behaviors after surgery. Identifying candidates for bariatric surgery with eating disorders prior to surgery and supporting them with specific ER-trainings could contribute to both patients’ satisfaction and lower costs and demands on the healthcare system.

The fact that there are no data providing information on binge eating disorder can be seen as a major limitation of the present study, considering that binge eating is known to be highly prevalent among obese individuals [26] and bariatric surgery candidates both pre- and post-surgery [30]. Binge eating disorder in turn is associated with higher rates of psychological impairment such as depression [42] and with markedly affected HrQoL [43], thus making its assessment and consideration even more relevant. As we were particularly interested in psychosocial variables, in the present study we mainly drew on self-report data. In addition, sample size was small and with the postoperative assessment taking place only 6 months after bariatric surgery, evaluation period was short, especially considering that “honeymoon phase” usually lasts about 2 years.

Clinical practice guidelines suggest a pre- as well as postoperative assessment by mental health professionals in order to identify patients who are likely to have difficulties adjusting to changes after bariatric surgery [44]. However, to date, postoperative psychological follow-up is not systematically provided all over Europe [45]. Screening for pathologic eating behaviors and depressive symptoms about 2 years after bariatric surgery when “honeymoon period” is over might prove especially useful. In this critical phase, weight loss has usually stabilized and patients begin to regain some weight [13], making an adaptive way of dealing with negative emotions especially relevant in order to prevent deterioration of mental well-being and significant increase of weight [46].
